# Bilateral Thalamic Infarction Due to Artery of Percheron Occlusion in a Young Patient With Hyperhomocysteinemia and Vertebral Artery Dissection

**DOI:** 10.7759/cureus.92282

**Published:** 2025-09-14

**Authors:** Norma Zuñiga Rivera, Carlos Ricardo Vazquez Sotelo, Andres Reyes Valdes, Alejandra Albarrán Sánchez

**Affiliations:** 1 Internal Medicine, Hospital of Specialties, XXI Century National Medical Center, Mexican Institute of Social Security, Mexico City, MEX

**Keywords:** artery of percheron infarct, bilateral thalamic infarction, hyperhomocysteinemia (hhcy), stroke in young population, traumatic vertebral artery dissection

## Abstract

The artery of Percheron (AOP) is a rare anatomical variant supplying the bilateral paramedian thalami and the rostral midbrain. Its occlusion represents a diagnostic challenge due to the variability in clinical presentation. We report the case of a 20-year-old male who was admitted with altered consciousness and nonspecific neurological symptoms. Magnetic resonance imaging (MRI) demonstrated a characteristic bilateral thalamic infarction consistent with AOP occlusion. Etiological workup revealed hyperhomocysteinemia and a right vertebral artery dissection, the latter suspected to be secondary to chiropractic cervical manipulation. Despite medical treatment and rehabilitation, the patient experienced significant cognitive and motor sequelae. This case highlights the importance of clinical suspicion and prompt diagnosis of AOP occlusion in young patients with atypical cerebrovascular events, emphasizing the need for a comprehensive evaluation to optimize management and improve prognosis.

## Introduction

The artery of Percheron (AOP) is an uncommon anatomical variant of the thalamic vasculature, consisting of a single arterial trunk arising from the P1 segment of the posterior cerebral artery that supplies both the paramedian thalami and the rostral midbrain [1]. Cerebral ischemic events involving the AOP territory are rare, with reported frequencies ranging from 0.1% to 0.3% of all strokes or even up to 0.53% in a single-center study [2]. Although the specific mean age for AOP infarction remains unclear, an early study noted that infarcts within the broader posterior cerebral artery (PCA) territory predominantly affect males, with a mean age of 61.5 years [3]. The clinical presentation of AOP infarction can be highly variable, potentially causing decreased level of consciousness and cognitive deficits, as well as ophthalmoplegia when it leads to rostral midbrain ischemia [4]. This variability contributes to diagnostic delays, particularly in younger patients where specific risk factors related to their sociodemographic profile must be considered [5].

Among the differential diagnoses that must be considered in a young patient with clinical signs of infarction are vascular causes (e.g., malformations) and vasculopathies, whether acquired or hereditary, such as Moyamoya disease and cerebral autosomal dominant arteriopathy-subcortical infarcts-leukoencephalopathy (CADASIL). Likewise, cardioembolic causes must be taken into account, for which the presence of a patent foramen ovale must be ruled out, as its identification and treatment can impact the patient's prognosis. Autoimmune and inflammatory disorders are also a cause of infarction in young adults, the etiologies of which can be vast. However, special attention should be paid to diagnoses such as systemic lupus erythematosus, sarcoidosis, or rarer entities like primary angiitis of the central nervous system, while always considering other factors that contribute to hypercoagulability [5]. Furthermore, drug use and a family history of hypercoagulability must be investigated; in this patient's case, all findings were negative.

## Case presentation

**Figure 1:**
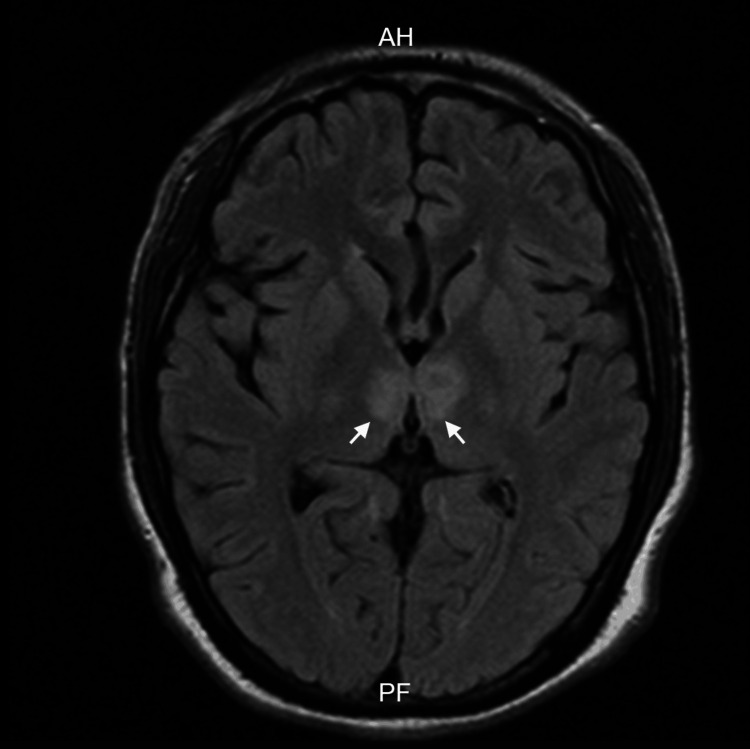
MRI FLAIR of bilateral thalamic infarction secondary to occlusion of the artery of Percheron Axial fluid-attenuated inversion recovery (FLAIR) demonstrates a bilateral hyperintense lesion located within both thalami. AH: Acute hemorrhage; PF: Posterior fossa.

**Figure 2:**
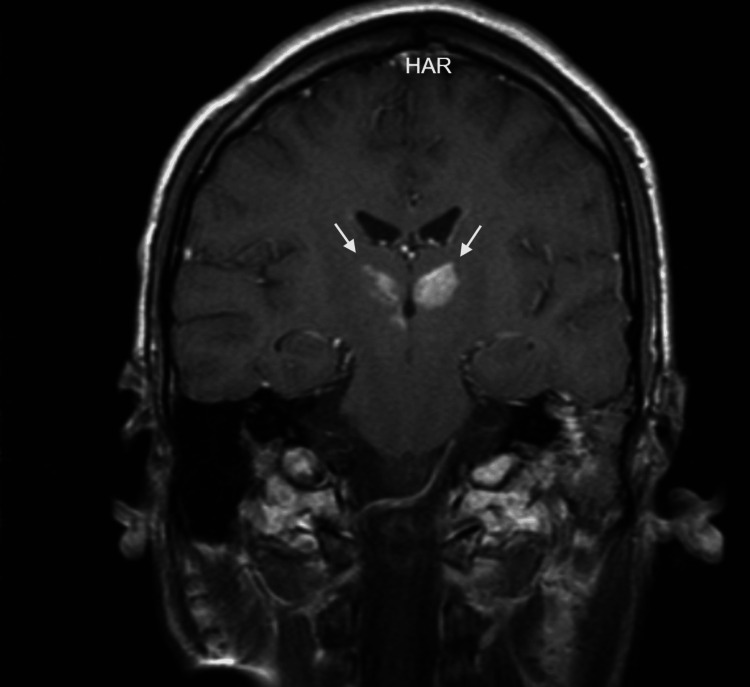
Coronal MRI of bilateral thalamic infarction secondary to occlusion of the artery of Percheron Coronal T1-weighted images were also acquired following the administration of gadolinium contrast. HAR: Holographic augmented reality.

A 20-year-old male, employed as an aluminum welder, with no relevant past medical history, presented with neck pain one week prior to lifting heavy objects at work, for which he underwent evaluation by a chiropractor involving cervical manipulation. On the day of admission, while asleep, he exhibited involuntary lower limb movements lasting over 30 minutes and was unresponsive to external stimuli. He presented to the emergency department with a Glasgow Coma Scale score of 6, temperature of 38.9°C, and positive Babinski, Kernig, and Brudzinski signs. Advanced airway management was performed, and he was admitted to the intensive care unit, where cerebrospinal fluid cultures were obtained, and broad-spectrum antibiotic treatment was initiated. Central nervous system infection was ruled out. An MRI study revealed bilateral thalamic infarction, affecting the AOP (Figures 1, 2).

He remained hospitalized for over a month due to nosocomial infectious complications and was discharged home with supportive measures (gastrostomy and tracheostomy). Further workup identified moderate hyperhomocysteinemia after an oral methionine load, positive markers of hemostatic activation, type 2 platelet hyperactivity, and a complete antiplatelet effect of acetylsalicylic acid (ASA). Thrombin/antithrombin complexes and prothrombin fragments 1+2 were increased. Platelet aggregometry induced with arachidonic acid was 2% (indicating adequate ASA effect). Cerebral angiography demonstrated an affected and filiform right vertebral artery, leading to the conclusion that the findings consistent with vertebral dissection. Treatment with ASA 150 mg daily was initiated.

At follow-up appointments 10 months later, bilateral third cranial nerve palsy of vascular etiology was evidenced. He also presented with neurological sequelae characterized by disorientation, deficits in working and anterograde memory, emotional lability, and easy tearfulness, with a Montreal Cognitive Assessment (MoCA) score of 17. Antiplatelet therapy was continued indefinitely.

## Discussion

The presented case involves a young patient who presented with an altered state of consciousness and was diagnosed with an ischemic stroke localized bilaterally in the thalamus, secondary to AOP occlusion. During the etiological workup for stroke in this young patient, two relevant factors that may have contributed to the event were identified: hyperhomocysteinemia and vertebral artery dissection.

The mechanism by which homocysteine causes endothelial damage has been postulated to primarily involve endothelial oxidative stress, inactivation of vasodilating agents such as nitric oxide, inactivation of protein C, inhibition of the fibrinolytic system, and activation of Factor V [6].

The clinical presentation of AOP infarctions is variable and may include (1) alterations in mental status, ranging from somnolence to coma, (2) behavioral changes, (3) aphasia and dysarthria, (4) ocular movement abnormalities, and (5) motor deficits, among other nonspecific manifestations [7]. Altered mental status, ocular movement disorders, and aphasia/dysarthria are the most frequently encountered clinical manifestations.

The workup of stroke in a young patient requires consideration of various less common etiologies compared to older populations. In this case, hyperhomocysteinemia was diagnosed, a metabolic disorder characterized by the systemic elevation of homocysteine, a thiol-containing amino acid. Epidemiological studies have indicated that hyperhomocysteinemia may increase the risk of stroke due to its impact on venous and arterial atherosclerosis, endothelial dysfunction, and vascular inflammation [8].

Additionally, a vertebral artery dissection was identified, a relevant etiology for stroke in young patients. The incidence of spontaneous vertebral dissection is estimated at 1-1.5 cases per 100,000 inhabitants, while traumatic dissection accounts for 0%-2% of trauma cases [9]. Numerous cases of arterial dissections have been linked to sudden stretching, although reports of dissection following cervical manipulation also exist. While a causal relationship between cervical manipulation and arterial dissection has not been definitively confirmed, some authors suggest it might act as a triggering or exacerbating factor for pre-existing vascular pathology, facilitating the development of a stroke [10]. Similar cases have been reported in which patients present with cervical or carotid artery dissection following manipulation in chiropractic activities. Although its pathophysiology has not been fully elucidated, a correlation is suspected between decreased elastin in the arterial walls and elevated homocysteine levels, an indirect consequence of homocysteine-induced activation of metalloproteinases [11].

The manifestation of third cranial nerve palsy can be explained by the infarcted area, as up to 53% of cases involve the paramedian thalamic region as well as the rostral midbrain, which in turn causes altered mental status accompanied by dysarthria, aphasia, and amnesia [12].

The most controversial aspect of this topic is the subsequent treatment for patients with hyperhomocysteinemia. The use of supplementation with folate, vitamin B12, and B6 remains controversial in the current literature, as the VITAmins TO Prevent Stroke (VITATOPS) trial showed no reduction in the number of events following supplementation [13]. Although antiplatelet agents have historically been recommended for secondary prevention in these patients, more studies are needed to establish a specific guideline on their benefit and long-term use. However, the multidisciplinary team performed a risk-benefit assessment and opted to continue long-term antiplatelet therapy due to the patient's high risk of recurrence.

## Conclusions

This case report underscores the critical importance of considering rare etiologies in young patients presenting with atypical stroke. AOP occlusion should be a key consideration in the differential diagnosis of young individuals exhibiting unexplained altered mental status or imaging findings of bilateral thalamic involvement. The identification of hyperhomocysteinemia and vertebral artery dissection as contributing risk factors in this patient further emphasizes the necessity of a thorough etiological evaluation in young stroke patients. Obtaining a detailed patient history, specifically inquiring about recent trauma or cervical manipulation, is paramount to identifying potentially reversible causes such as arterial dissection and ultimately optimizing patient outcomes. Furthermore, it highlights the importance of conducting studies that can clarify an optimal treatment for patients with hyperhomocysteinemia, as no consensus currently exists.
